# Occupational challenges of young adult patients with congenital heart disease

**DOI:** 10.1007/s12471-014-0540-1

**Published:** 2014-02-22

**Authors:** M. A. Sluman, S. de Man, B. J. M. Mulder, J. K. Sluiter

**Affiliations:** 1 Department of Cardiology, Academic Medical Center, Amsterdam, the Netherlands; 2 Interuniversity Cardiology Institute of the Netherlands, Utrecht, the Netherlands; 3 Coronel Institute of Occupational Health, Academic Medical Center, Amsterdam, the Netherlands; 4 Department of Cardiology, Academic Medical Center, Room B2-215, Meibergdreef 9, 1105 AZ Amsterdam, the Netherlands; 5 Department of Cardiology, Academic Medical Center, Room B2-240, Meibergdreef 9, 1105 AZ Amsterdam, the Netherlands

**Keywords:** Congenital heart disease, Work, Employment, Qualitative research

## Abstract

**Background:**

Despite improved survival of adults with congenital heart disease (CHD), higher rates of unemployment and work-related problems are seen, especially among younger adults. This study was performed to gain insight into current barriers and facilitating experiences at work among young adult patients with CHD.

**Methods:**

This qualitative study consisted of semi-structured face-to-face interviews, based on a self-constructed model from several existing models, which were held among outpatients with CHD from a large tertiary referral centre. Verbatim transcribed audio-taped data were analysed using a directed model-based content analysis approach.

**Results:**

Fifteen patients had been interviewed when data saturation was reached. Work was important for all participants. Several barriers and facilitating factors were identified. Barriers were mostly on physical aspects and lack of opportunities for recovery. Important facilitating factors were good relationships with colleagues and employer and having sufficient opportunities for recovery. Most of these factors are also seen among patients with other chronic diseases, but with a different priority.

**Conclusion:**

This is the first study that has identified qualitative factors at work of young adult CHD patients. Work is important to them. Challenges are dealing with the physical barriers and getting enough support from colleagues. Specific coaching or a tailored group intervention could thereby be helpful. Future research should aim at the aetiology of problems and identifying patients who would benefit most from specific coaching.

## Introduction

The number of patients with congenital heart disease (CHD) who reach adulthood has grown extensively over the last decades. Due to the great development in techniques, CHD has more and more become a chronic disease [1, 2]. Aspects such as education and work therefore become increasingly important and are sometimes even more important contributors to quality of life than health status itself [3, 4].

Socio-economic outcomes among adults with chronic diseases are far from optimal, with unemployment rates nearly twice as high as among healthy individuals [3]. Several studies have identified factors contributing to maintaining work among different kinds of chronic diseases such as rheumatoid arthritis, asthma and ischaemic heart disease [3, 5]. Previous studies have shown that most patients with CHD are able to work, although higher rates of unemployment are seen in most observational studies compared with the general population, especially among severe CHD [6–10]. Physical problems are often reported as a reason [11]. A recent study from our group showed that patients with (even mild) CHD, especially patients under the age of 40 years, were more often unemployed, worked less hours and had lower incomes than their healthy peers [8]. Although socio-economic outcomes in CHD need to improve, specific information on the experiences and needs of employees with CHD at work is lacking. Therefore, this study aims at exploring barriers and facilitating factors adults with CHD experience at work. This will help us gain better understanding and suggest possible strategies on how to intervene.

## Methods

Qualitative research was conducted to explore experiences at work among young adult patients with CHD through semi-structured in-depth face-to-face interviews [12]. The study followed the ethical recommendations of the Declaration of Helsinki [13]. COREQ criteria were used as the fundament for reporting the data [14].

### Instruments and procedures

The structure of our self-developed interview was based on a model we constructed from several existing models, based on a theoretical framework (Table 1). This framework consisted of a combination of the classical ‘Workload - work capacity model’ [15], the ‘Effort and recuperation model’ [16], the ‘Structure of the psychosocial work environment model’ of the QEEW (Questionnaire Experience and Evaluation of Work) [17] and information from qualitative studies in other patient populations . Barriers that were described in the literature were mostly on physical job demands, low social support and fatigue [5]. We added specific items for patients with CHD that are known from the literature, such as the feeling of being different, revealing or hiding the CHD and coping with the disease and the healthcare system [18, 19].

**Table 1:**
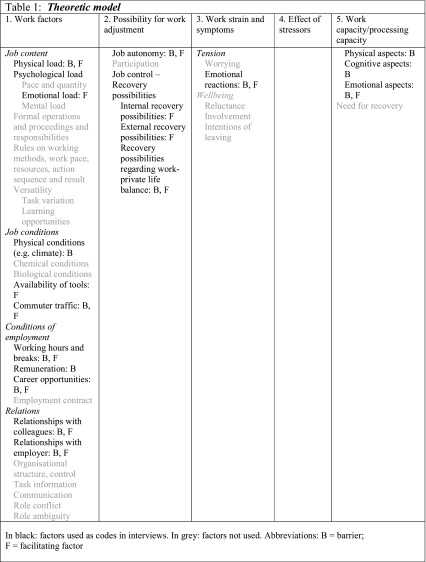
Theoretic model

The interviews consisted of questions on demographic items, including information about education and the contents of the patient’s current work, working times, type of contract and job choice. Besides this, experienced barriers as well as facilitating factors at work in relation to the CHD were the main subject of the interview. During the interview, participants were encouraged to speak openly to gain more in-depth information. To improve reliability, the interviewer recapitulated important parts of the answers given during the interview for member checking. A pilot interview was performed to pre-test the usability of the interview with feedback from an experienced interviewer. The interviewer (SdM) had no relations with the selected patients and was not directly involved in their care.

Patients were screened between February and April 2012 through information in their medical files. All patients were treated in one large outpatient cardiology clinic from a tertiary referral centre in the Netherlands. Patients were eligible if they had known CHD, they were in paid work or had been in paid work in the past 6 months (following the definition of Statistics Netherlands), had a full understanding of the Dutch language and were aged between 20 and 35 years. Patients with mental retardation or severe comorbidity were excluded. Heterogeneity in CHD severity, age, gender, jobs and clinical classification according to the New York Heart Association (NYHA) was intended. CHD severity was based on a consensus-based classification scheme [20].

An information letter was sent to selected patients in advance. Patients were approached to participate after their regular appointment at the outpatients clinic. Informed consent was obtained verbally. All interviews were held in Dutch (the native language for both interviewer and interviewees) and were audio-taped. The interviews lasted between 18 and 56 min (mean duration 34 min). All interviews but one were held in a private room of the outpatient clinic. One interview was, on request, held at the participant’s home. In five interviews a partner or parent of the participant was present and able to contribute to the interview. All supported the patient’s opinions.

### Data analysis

All interviews were transcribed verbatim and analysed with the directed content analysis approach by two researchers (SdM and MS) [21]. This analysis approach consists of identifying meaningful text fragments, selective open coding and interpretation. Our own model was used as a frame for coding the data (Table 1). After coding, analyses were compared and differences were discussed until consensus was reached. To increase reliability of the analysis, three interviews were read and open coded by a third researcher (JS). Data saturation was checked during the process and reached after participant 14. Negative case analyses consisted of discussion within the research team. Illustrative quotations were selected from different interviews by the research team. MAXqda 10 software [Kuckarts, Udo. Berlin, Germany] was used for analysis.

## Results

### Participants

In total, 15 patients were interviewed between April and May 2012. Twenty-four patients were preselected and asked to participate. Patients who did not participate did not have time (7 patients) or did not fulfil the criteria (2 patients did not work).

Eight patients were male and the mean age of the participants was 28 years (range 22–35 years). Demographic and work characteristics are described in Table 2. Three participants were not working or worked less hours due to recent surgery, but had been working in the past 6 months. With these participants, working experiences were discussed from the period before and after surgery. One patient with comorbidity was not excluded because he did not experience any limitations in his very demanding job and could therefore contribute important information. All participants attended at least secondary school. CHD had affected job choice in 5 participants. One participant performed a less physical job than he desired. The other 4 participants worked at a lower level than their educational level. Overall, work played an important role in the daily lives of all participants. Two participants, with mild and moderate CHD and NYHA I, experienced neither barriers nor facilitating factors at work that could be related to their CHD. Two participants with moderate CHD and NYHA I only experienced facilitating factors. The other 11 participants, all NYHA II but with ranging severity of CHD, experienced barriers and facilitating factors. No participants experienced only barriers. Tables 3 and 4 show all reported factors.

**Table 2:** Demographic, work and clinical characteristics

Participant	Sex	Age	Diagnosis	Severity	NYHA	Type of work	Operation, remaining defect	Hrs work/week
1	F	23	PS	Moderate	II	Nursing student	Balloon pulmonary valvuloplasty at age 9; remaining mod. PR and PS	38 – 40
2	M	26	CoA, BAV	Moderate	I	Gardener	Corrected through subclavian flap at age 6; BAV with good function	45 – 54
3	M	29	Supravalvular AS	Moderate	II	Administrative assistant	Corrected in childhood	38
4	M	22	ToF	Moderate	I	Team leader	Corrected at age of 2; homograft at age 22, remaining mild PR and PS	40
5	F	27	Subvalvular AS	Moderate	I	PhD candidate	Mild AR and AS	50
6	M	29	TGA	Severe	II	Window cleaner	Arterial switch in first year; patch in RVOT because of PS at age 20, remaining mod. PR; systemic LV with good function	21
7	M	24	ToF	Moderate	II	Laboratory technician	Corrected in first year; homograft at age 17, remaining mild PR and mod. PS	32
8	F	27	BAV with sever AS, dilated root	Moderate	II	Day care centre	Aortic root replacement at age 27 (recent)	32
9	F	35	TGA, VSD, overriding aorta	Severe	II	Hairdresser	Arterial switch and closure of VSD at age 2. Bentall surgery because of severity. AR at age 34. Decreased LV and RV function	25
10	F	32	Pulmonary atresia, VSD	Severe	II	Sales assistant in parents’ fish shop	Patch in RVOT at age 2; closure of VSD at age 4; severe PR, PAH, ventricular tachycardia	16
11	F	32	ccTGA, VSD, PS, situs inversus, dextrocardia	Severe	II	Management assistant	VSD closure at age 3, pacemaker implantation due to postoperative AV-block; balloon valvuloplasty; systemic RV; mild PR, mod. PS	15
12	M	24	BAV with severe AR	Mild	II	Administrative assistant	Bentall surgery at age 24 (recent)	40
13	M	28	Interrupted aortic arch, VSD	Moderate	II	Excavation worker (digger)	Both corrected in first year; re-operation at age 9 with Dacron bypass; bronchus obstruction from bypass at age 19	40
14	F	34	Tricuspid atresia, VSD, ASD 2, PS	Severe	II	Receptionist	Palliated at age 1 by central aortic-pulmonic shunt, Fontan surgery at age 6, re-operated at age 18 and 34 (recent)	12
15	M	26	ASD 2	Mild	I	PhD candidate	Surgical closure at age 2. Remaining RV dilatation	60

*Abbreviations*: *F* female, *M* male, *NYHA* New York Heart Association classification, *PS* pulmonary stenosis, *PR* pulmonary regurgitation, *CoA* coarctation of the aorta, *BAV* bicuspid aortic valve, *AS* aortic stenosis, *ToF* tetralogy of Fallot, *AR* aortic regurgitation, *TGA* transposition of the great arteries, *RVOT* right ventricular outflow tract, *LV* left ventricle, *RV* right ventricle, *PAH* pulmonary arterial hypertension, *ccTGA* congenitally corrected transposition of the great arteries, *VSD* ventricular septal defect, *ASD 2* atrial septal defect type 2

**Table 3:** Observed barriers

	Participants														
Barriers	1	2	3	4	5	6	7	8	9	10	11	12	13	14	15
1. Work factors															
*Job content*															
**Too much physical load**	•					•	•	•			•		•		
*Job conditions*															
**Heavy physical conditions (e.g. climate)**						•				•	•	•	•		
Negative effects of commuter traffic								•						•	
*Conditions of employment*															
**Too much working hours, too little breaks**						•				•	•			•	
Too low remuneration						•									
**Too little career opportunities**						•					•	•		•	
*Relations*															
**Bad relationships with colleagues**														•	
**Bad relationships with employer**						•			•		•				
2. Possibility for work adjustments															
Too little job autonomy									•						
Job control – Recovery possibilities															
**Too little recovery possibilities to preserve a good work-private life balance**	•									•	•			•	
3. Work strain and symptoms															
*Tension*															
Negative emotional reactions						•			•		•				
4. Negative effect of stressors						•						•			
5. Work capacity/processing capacity															
**Negative physical aspects**	•		•			•	•	••		•	•		•	•	
**Negative cognitive aspects**	•						•				•	•			
Negative emotional aspects						•		•	•		•				

• = code observed in interview of participant; in bold = codes described in results

**Table 4:** Observed facilitating factors

Facilitating factors	Participants														
	1	2	3	4	5	6	7	8	9	10	11	12	13	14	15
1. Work factors															
*Job content*															
**Right amount of physical load**			•	•		•	•			•				•	
Psychological load															
Positive emotional load									•						
*Job conditions*															
Availability of tools								•			•				
Positive effects of commuter traffic							•							•	
*Conditions of employment*															
**Right amount of working hours and breaks**						•	•	•		•	•				
**Having career opportunities**											•			•	
*Relations*															
**Good relationships with colleagues**			•	•	•	•	•	•	•	•	•		•	•	
**Good relationships with employer**				•	•	•	•				•			•	
2. Possibility for work adjustments															
**Enough job autonomy**						•	•			•	•				
Job control – Recovery possibilities															
**Enough internal recovery possibilities**	•					•	•	•	•	•		•	•		
**Enough external recovery possibilities**						•	•	•			•			•	
**Enough recovery possibilities to preserve a good work-private life balance**						•	•			•		•		•	
3. Work strain and symptoms															
*Tension*															
Positive emotional reactions														•	
4. –															
5. Work capacity/processing capacity															
Positive emotional aspects											•		•		

• = code observed in interview of participant; in bold = codes described in results

### Barriers

The factors that were most frequently mentioned as barriers for work functioning are described below (Table 3).

#### Physical aspects

Participants often experienced the physical load of their work as too high, frequently leading to being less able to perform their job correctly. When working in a warm environment (heavier physical condition), multiple participants experienced problems of tiredness and complaints of dyspnoea.

[…when my colleagues think it is just warm outside, I get really short of breath…] [Participant 6, P6]

#### Lack of opportunity for recovery and processing capacity

Most participants had problems with the balance between workload and the physical processing capacity they need for the job. Some of the participants experienced too little opportunity for recovery to preserve a good balance in work and private life, due to their increased need for recovery after work. This was mostly expressed as being fatigued during a working day. Fatigue was often more present after working several days in a row and was often experienced to be more present than in their healthy colleagues. A barrier mentioned by several participants was hours of work or working several days in a row. For some patients working full time was not possible, because they were not able to recover enough before returning to work.

[…as the day progresses I get tired quite quickly. Even though I do seated and not very physical work, I get tired very quickly anyway.] [P7]

Some participants had a problem with the balance between workload and the cognitive processing capacity needed for the job. When workload was too high or processing capacity too low, participants experienced a decreased concentration ability during their workday.

[… than you will read a sentence two, three times. And you just do not get it, you just do not take it in fully. That is when I think: ‘I’ll have to leave it for another day.’] [P1]

#### Relationships with employer

Problems were experienced with employers when they could not understand the consequences of CHD for their employees. Therefore, some participants had been forced to transfer to a different job. Another patient had to arrange most of her rehabilitation at work after surgery without the support of her employer. For most participants these experiences were difficult to go through. Some participants experienced problems in their career opportunities. Applying for a job was difficult for some participants, partly due to physical limitations as well as experienced social disadvantage. Due to this, four participants worked at a lower level than their educational level. For most of the patients, the decision to tell the employer about their CHD had been difficult and well-considered.

[…At that time I noticed that it was difficult to apply…usually I was too honest about my CHD and then you would not get the job.] [… I can actually do a lot more, so that’s a shame.] [P11]

### Facilitating factors

Facilitating factors for work functioning with CHD are mentioned in Table 4 and the most important factors are described below.

#### Physical aspects

Some facilitating factors that were reported are not surprising in view of the barriers mentioned before. Participants benefitted, for example, from doing less physical work. Working at an office instead of having a demanding physical job, having better working hours or working less hours a day or a week or interrupting the work week by a day off were all mentioned as facilitating.

#### Opportunities for recovery

Almost all factors in the theory model that were placed under possibility for work adjustment were recorded as important in facilitating. Job autonomy (being able to decide which task you do when and how) was observed as very important in several interviews.

[I work in a fish shop. But when it’s warm we just don’t fry the fish, since I already feel short of breath then.] [When I need to carry something, there is always someone who does it for me.] [P10]

Internal recovery opportunities, for example to be able to take a break or to rest during work when needed or being able to adjust the working environment, as well as external recovery opportunities, such as being able to recover during free time, for instance by taking a day off, were mentioned as helpful.

[If I indicate: ‘Well, it is too much, I need 2 days off.’ Then it is quite easily arranged.] [P7]

Having enough opportunity for recovery to preserve a good work-private life balance was also a returning subject. Participants needed more time, more than colleagues they believed, to recover from work in order to have enough time to live their private life’s.

[…Sometimes when it’s worse, you feel really tired for a while. Then they say: ‘That’s fine. Go home early or change your day’.] [P11]

#### Relationships with colleagues and employer

Good relationships with colleagues was experienced as a facilitating factor among almost all participants. Colleagues helped participants with their work or took over when needed. Participants also felt supported when colleagues showed interest in them and (the consequences of) their CHD. Involvement through a period of sick leave was considered very supporting. A good relationship with the employer was seen as facilitating, based on support and flexibility of the employer, for example by letting them perform less physical work or letting them work less or flexible hours. How easily employees could arrange to stay away from work for hospital visits was very different among participants.

Though having too few career opportunities was reported as a barrier, some participants experienced benefit from their CHD in getting a job. One participant for example was hired after applying for a vacancy specifically aimed at occupationally handicapped persons.

## Discussion

This is the first study that has identified both barriers and facilitating factors at work among young adults with CHD. Important facilitating factors were good relationships with colleagues and having enough recovery possibilities. Barriers were mainly caused by physical aspects and too little opportunity for recovery.

Work is important in the daily lives of young adults with CHD, and it is influenced by their CHD. CHD affects job choices and work in many ways: from adjustments that have already been made to prevent possible future problems to having been forced to change jobs due to their illness. Even though some of the relatively young adults with CHD experienced no or few problems at work, the disease influenced job choice in several patients and a substantial number of them worked below their educational levels. Considering that average educational levels among people with CHD are already a little below the general population [8], this is a serious issue that can lead to loss of talent and labour forces.

Factors that are described by this population of young adults with CHD are partly in accordance with what is seen by other patients with chronic diseases [22], although their weight seems somewhat different. A lot of barriers that were found among employees with CHD can be summarised to be caused by physical aspects and recovery capabilities and opportunities. Fatigue, decreased concentration and exertional dyspnoea were mentioned most. In this population, specific pathways could play a role. A lot of late complications of CHD present with fatigue [23]. This may also be influenced by the use of certain medications. Furthermore, patients with CHD are at increased risk for neurological, psychological and cognitive damage through many pathophysiological ways, especially those with severe or cyanotic defects [24]. Whether this is due to brain damage or cognitive impairment by the treatment or the CHD itself or is caused by something else is as yet unknown. Especially among adults with CHD there is very little information about the relationship between possible neuro-cognitive damage and practical outcomes. However, this does not explain why several participants worked at a lower level than their educational level. Previous studies among healthy individuals have shown that the need for recovery during and after working time is a major predictor of psychosomatic symptoms, sleep problems, and complaints of emotional exhaustion. This could lead to a vicious circle of more fatigue and physical problems, even potentially leading to sick leave.

Significant facilitating factors were mostly about good relationships with colleagues and employers and about having a sense of control: experiencing opportunities to adjust how and when the work was performed and to preserve an optimal work-private life balance. In the facilitating factors we might find the key to the solution: flexibility in work conditions and arrangements for breaks as well as positive relationships with colleagues and employers seem essential. Varekamp et al. have looked at workplace problems and solutions for people with different kinds of chronic diseases and found that working less hours, working at home, a slower work pace and more job autonomy were most desired solutions by patients with chronic diseases [25]. Several studies also show that motivation, personal coping strategies and personality traits are important for work participation for employees with other chronic diseases [27]. Showing interest or active support is highly appreciated and necessary: a lot of people with CHD who are considered to be asymptomatic report problems on health questionnaires [26]. Previously reported experiences of young adults with CHD also pointed out that it is hard to strike a balance between being a patient or a ‘regular’ employee (being different or not) and that there is often some ambivalence about telling people about the CHD [19]. This issue was also brought up by several participants in this study.

The results of this study can be used to tailor a specific (group) self-management intervention program to support CHD patients in their work [27]. Previous studies on these kinds of intervention programs for other chronic diseases show a decrease in physical symptoms and fatigue and better coping strategies [28, 29]. Specific coaching for patients should try to increase self-confidence, assertiveness and the sense of job control and thereby consist of acquiring skills in how to deal with specific physical symptoms, to increase job autonomy, focus on job motivation and give insight into personal coping strategies and personality traits that could be of influence. Given our results, it should also emphasise the importance of good relationships with colleagues and employer. Considering certain CHD-specific aspects, for example telling colleagues and employer about the CHD and dealing with specific physical symptoms, a specialised intervention program for CHD patients could fit all of these needs. For employers and career advisors, helping patients with CHD should focus on reducing high physical job demands, especially for patients with more severe defects, and offering possibilities to arrange adaptations in the workplace and work hours when needed.

### Study limitations

Although qualitative research is relatively uncommon and unknown within the field of clinical cardiology, we believe this is the best way to gain a better understanding of this subject. In the interpretation, however, since this is a qualitative study, we must realise that explanations for the higher rates of unemployment or work-related problems or direct solutions are not to be expected.

A shortcoming of the study is that, in hindsight, heterogeneity in patients functional class was not completely reached. Only patients with NYHA I and II were interviewed and this may have underestimated the barriers for work among patients with CHD and a lesser functional status. On the other hand, no patients with mild CHD were included. A limitation that may also have underestimated the barriers is that by the inclusion criteria of having a job, people with potentially the most or most severe barriers could have been excluded. A strong aspect of this study is that data were gathered through the use of a specific model. This theoretical model could, due to its basics, therefore include all relevant factors in one model. Because participants were able to talk openly and because the directed content analysis approach was used, no factors seem to be overlooked.

## Conclusions

It is important for CHD patients to arrange their work in such a way that it is possible for each specific employee to function properly. To achieve this, a committed employer and employee are required. Possibilities for making personal adjustments in tasks or schedules and a good relationship with colleagues and employer play a pivotal role here. Healthcare professionals should be more aware that CHD can lead to specific barriers in the workplace. Further research on the neuro-cognitive background and aetiology of problems of concentration and fatigue may improve these barriers on the work field for long-term survivors of CHD and may help develop specific intervention programs and identify patients who would benefit most.
